# What can other animals tell us about human social cognition? An evolutionary perspective on reflective and reflexive processing

**DOI:** 10.3389/fnhum.2012.00224

**Published:** 2012-07-27

**Authors:** E. E. Hecht, R. Patterson, A. K. Barbey

**Affiliations:** ^1^Graduate Neuroscience Program, Emory University, AtlantaGA, USA; ^2^Yerkes National Primate Research Center, Emory University, AtlantaGA, USA; ^3^Center for Translational Social Neuroscience, Emory University, AtlantaGA, USA; ^4^Department of Philosophy, Emory University, AtlantaGA, USA; ^5^Decision Neuroscience Laboratory, University of Illinois at Urbana-Champaign, ChampaignIL, USA; ^6^Beckman Institute for Advanced Science and Technology, University of Illinois at Urbana-Champaign, UrbanaIL, USA; ^7^Department of Internal Medicine, University of Illinois at Urbana-Champaign, ChampaignIL, USA; ^8^Department of Psychology, University of Illinois at Urbana-Champaign, ChampaignIL, USA; ^9^Department of Speech and Hearing Science, University of Illinois at Urbana-Champaign, ChampaignIL, USA; ^10^Neuroscience Program, University of Illinois at Urbana-Champaign, ChampaignIL, USA

**Keywords:** reflective processing, reflexive processing, social cognition, empathy, comparative cognition, evolution, motor resonance

## Abstract

Human neuroscience has seen a recent boom in studies on reflective, controlled, explicit social cognitive functions like imitation, perspective-taking, and empathy. The relationship of these higher-level functions to lower-level, reflexive, automatic, implicit functions is an area of current research. As the field continues to address this relationship, we suggest that an evolutionary, comparative approach will be useful, even essential. There is a large body of research on reflexive, automatic, implicit processes in animals. A growing perspective sees social cognitive processes as phylogenically continuous, making findings in other species relevant for understanding our own. One of these phylogenically continuous processes appears to be self-other matching or simulation. Mice are more sensitive to pain after watching other mice experience pain; geese experience heart rate increases when seeing their mate in conflict; and infant macaques, chimpanzees, and humans automatically mimic adult facial expressions. In this article, we review findings in different species that illustrate how such reflexive processes are related to (“higher order”) reflexive processes, such as cognitive empathy, theory of mind, and learning by imitation. We do so in the context of self-other matching in three different domains—in the motor domain (somatomotor movements), in the perceptual domain (eye movements and cognition about visual perception), and in the autonomic/emotional domain. We also review research on the developmental origin of these processes and their neural bases across species. We highlight gaps in existing knowledge and point out some questions for future research. We conclude that our understanding of the psychological and neural mechanisms of self-other mapping and other functions in our own species can be informed by considering the layered complexity these functions in other species.

## Introduction: how can an evolutionary perspective inform human neuroscience?

Students of evolutionary neuroscience may be familiar with the metaphor of an old apartment building for brain evolution. At first, the building is heated by a series of wood-burning fireplaces. Later, a coal-fueled steam system is added in the chimneys and hearths. Later still, an HVAC system is installed, with electrical wiring grafted to the old hot water pipes. Every time something goes wrong with the heat, someone has to determine whether the problem is due to a wiring problem in the HVAC system, damage to the old hot water pipes along which those wires run, or a structural problem in the old chimneys that house the whole apparatus. Like the addition of new heating systems to the apartment building, evolution adds new functions to the brain by building on the pre-existing architecture. Thus the old systems don't disappear: their new functions are integrated with their pre-existing ones, and the continued function of the new systems relies on the soundness of the old ones.

A prominent instantiation of this idea was MacLean's triune brain theory (MacLean, 1990), which posited that instinctual behavior is controlled by the brain's “reptilian complex” (basal ganglia), basic social behavior by the “paleomammalian complex” (limbic system), and higher cognitive function by the “neomammalian complex” (cerebral neocortex). Later anatomical work showed this model to be overly simplistic, but the basic concept of hierarchical processing is echoed by the recent proliferation in dual process models in neuroscience and psychology. Current models tend to make a two-way distinction. One type of system is described as unconscious or preconscious, implicit, automatic, low effort, rapid, perceptually driven, while another is described as conscious, explicit, controlled, high effort, slow, and analytic or reflective [reviewed in Evans (2008)]. For the sake of simplicity, we will refer to the first sort of system as “reflexive” and the second as “reflective,” although this two-way distinction is likely also overly simplistic.

For some time, it was assumed that reflective social cognitive processes were evolutionary “upgrades” unique to humans, or perhaps humans and our closest living relatives, and much behavioral research focused on identifying which skills are “uniquely human” (Evans, 2008; de Waal and Ferrari, 2010). However, there are reasons not to assume that humans' most advanced forms of social cognition lack correlates in other species. Like the upgrades to the apartment heating system, human social cognitive “upgrades” must achieve the same basic purpose as their simpler predecessors—interacting with other individuals in the environment in an adaptive way. Evolution modifies previously existing forms to create new ones (for example, wings are modifications of limbs), and the new forms retain some features of the old ones (bone structure). These adaptations must arise in the context of a previously working social cognitive system, and as such, must incorporate with it. New neural mechanisms must function within the organism's existing social cognitive framework, or else the organism's social behavior will be impaired and its chances of survival will be reduced. Therefore, neural adaptations for new social cognitive functions are likely to involve some of the same neural architecture as preexisting systems.

Furthermore, functions that were once attributed only to humans are increasingly being identified in other species. Thus, reflective social cognition is probably uniquely *developed* in humans, but not unique *to* us (Evans, 2008). It is important to remember that all life on earth has been evolving for the same amount of time and the phylogenic tree has no “top.” Differences in function represent adaptation to different niches, not higher or lower position in a *scala naturae*. A growing number of researchers in the field of comparative behavior stress the explanatory utility of viewing most behavior as phylogenically continuous (de Waal and Ferrari, 2010), a position that was espoused by Darwin (1872).

All of this argues that studying animals can tell us something about human social cognition. Human neuroscience is currently very interested in the brain's “most modern upgrades”—reflective processes like theory of mind, or thinking about what another person is thinking (Premack and Woodruff, 1978), as well as related processes like imitation, perspective-taking, and empathy. Understanding these functions is relevant for understanding and treating disorders of social cognition like autism in which they are impaired. But like the heating in the old apartment building, these functions aren't stand-alone systems. Deficits in the higher level functions may even be due to underlying, less obvious deficits in the lower level functions. In such cases understanding the interplay between higher- and lower-level functions is essential for understanding and treating deficits and disease affecting higher level social functions.

In this review, we explore the interplay between higher- and lower-level functions, as well as the question of what in particular the study of animals can tell us about human social cognition. We do so in the context of *self-other matching*, defined as any phenomenon in which the observation of another's behavior or state causes the observer's behavior or state to become congruent with it. We have chosen this domain for several reasons. First, the operational definition allows phenomena to be categorized by easily observable output. In many species, comparable behavioral data is available but data about underlying physiology or neural substrates is not (or it is available but contentious, as in the question of whether human imitation involves or relies on the mirror system). Grouping results by behavioral output allows for cross-species comparisons without any a priori perspective about underlying physiological processes. We will, however, draw connections to underlying physiological and neural substrates when possible. Second, self-other matching can occur in a reflexive manner, but this reflexive processing can have measurable effects on reflective processes. Third, self-other matching phenomena are present in varying degrees of complexity across a wide range of phyla. In this review, we limit our scope to vertebrates. We focus heavily on primates, since they are most closely related to humans and also the subject of a large body of comparative research, but we also discuss some research in canids, rodents, birds, and reptiles. In humans, self-other matching encompasses phenomena like motor resonance, mimicry, imitation, emulation, empathy, and perspective taking (defined in Table 1), which likely rely on partially discrete and partially overlapping neural and psychological mechanisms. Comparing which of these functions are present in which other species can help us to structure our thinking about the organization of these processes within our own species.

**Table 1:** **Terms and definitions**.

**General terms**	Mimicry	In this review, used as a general, non-specific umbrella term for any kind of reflexive, non-intentional, overt self-other matching
	Copying	In this review, used as a general, non-specific umbrella term to refer to any kind of intentional, reflective, overt self-other matching
**Motor domain**	Motor resonance	Activation of common neural or psychological substrates for observed and executed action—e.g., observing another's action causes my motor system to “resonate” with theirs
	Motor contagion	The overt, reflexive mimicry of an observed action via motor resonance
	The “chameleon effect”	Humans' tendency to reflexively mimic others' postures, mannerisms, facial expressions, and behaviors, which plays a functional role in human social interactions
	Motor interference	A reduction in movement accuracy when observing a non-congruent movement, caused by reflexive motor resonance
	Social learning or observational learning	Family of mechanisms by which an individual can copy an observed goal-directed behavior
	Emulation	Copying an action's goal or end result, but not its component movements or methods
	Imitation	Copying both an action's end result and the component movements
	Overimitation	Copying component movements which do not contribute to reaching the action's goal
**Perceptual domain**	Gaze following	A shift in eye gaze direction in order to match one's own visual perception to another individual's
	Following gaze geometrically	Following another individual's gaze behind a barrier; inferred to imply the ability for perspective-taking
	Perspective taking	The understanding that another's perceptual knowledge can differ from one's own (not always used to connote a reflective process)
	Theory of mind	The understanding that another's representational mental states can differ from one's own (a type of perspective taking; generally connotes a reflective, controlled process)
**Autonomic/emotional domain**	Contagion	The reflexive instantiation of an observed emotional or autonomic state in one's self (non-referential)
	Observational fear learning	Acquiring a fear response to a particular stimulus based on observation of another individual's experience with that stimulus (referential)
	Rapid facial reactions	Brief, reflexive, low-intensity mimicry of observed facial expressions, measurable by increased EMG activity in congruent facial muscles
	Cognitive empathy	A referential, reflective, explicit understanding of another individual's emotional state

## Experimental results: a compendium of self-other matching phenomena across species

### Self-other matching in the motor domain: somatomotor movements

Somatomotor self-other matching can occur at a reflexive level via motor resonance. Motor resonance is a general idea implicating the activation of common neural or psychological substrates for observed and executed action—e.g., observing another's action causes my motor system to “resonate” with theirs. When motor resonance causes the overt output of an observed action, this is termed “motor contagion”. A well-known example of motor contagion occurs during infancy. For a brief period in development, neonatal macaques, humans, and chimpanzees copy observed orofacial movements (Meltzoff and Moore, 1977, 1983; Heimann et al., 1989; Myowa-Yamakoshi et al., 2004; Ferrari et al., 2006; Bard, 2007; Ferrari et al., 2009a,b; Paukner et al., 2011). Human infants also copy observed finger movements (Nagy et al., 2005). This effect disappears sometime around age 2 weeks in macaques, 2 months in chimpanzees, and 3 months in humans (Meltzoff and Moore, 1977, 1983; Heimann et al., 1989; Myowa-Yamakoshi et al., 2004; Ferrari et al., 2006). The fact that this period lasts longer in humans may be relevant to species differences in adult social cognition, although this idea awaits exploration. In adult humans, motor contagion in everyday social interactions is sometimes called the “chameleon effect”—the tendency to mimic others' postures, mannerisms, facial expressions, and behaviors. It increases liking, smoothes social interactions, and is more common in empathic people (Chartrand and Bargh, 1999). Orangutans spontaneously and rapidly mimic facial expressions during play (Davila Ross et al., 2008), chimpanzees experience contagion for aggressive and affliative social interactions (Videan et al., 2005), and macaques are more likely to eat when seeing or hearing another monkey eat (Ferrari et al., 2005). In Paukner et al. (2011), human experimenters imitated capuchin monkeys' actions on a ball, such as poking or mouthing it. The monkeys later preferred to spend more time in proximity to imitator versus non-imitator humans, and also preferred to interact with them in a task where tokens could be exchanged for food. This suggests that motor contagion may play a role in their naturalistic social interactions and may be important for establishing affiliative relationships and prosocial behavior.

In addition to facilitating the production of actions congruent to others', motor resonance can interfere with the production of non-congruent actions. This is termed “motor interference” and is measured by a reduction in movement accuracy while observing a non-congruent movement. In humans, motor interference appears around age 4–5, is influenced by prior knowledge or experience of the individual performing the observed action, is weakened by self-focus, and is stronger when the subject has practiced the observed action and when the demonstrator is similar to the subject (Marshall et al., 2010; Saby et al., 2011). Observing a sinusoidal arm movement interferes with the observer's own movement more if the observed movement is directed toward a goal, suggesting that goal directed actions are more contagious than non-goal-directed actions (Bouquet et al., 2010). To our knowledge, motor interference has not been studied in other species, although like motor resonance, it seems to be an easily addressable topic. For example, in a paradigm used to study reach-to-grasp movements, macaque monkeys grasp a bar in an apparatus that measures the force, velocity, and direction of their arm movements [e.g., (Kalaska et al., 1989)]. This could be used to measure perturbations to a monkey's movements while watching congruent versus incongruent movements by another monkey.

In humans, evidence for a shared physiological basis of action execution and observation at a low level comes from electrophysiological experiments. Transcranial magnetic stimulation (TMS) to motor cortex can be used to produce motor-evoked potentials (MEPs) in the periphery—e.g., stimulation to the thumb area of primary motor cortex evokes a measurable electrophysiological effect in the thumb muscles. In these TMS experiments, MEPs are greater during observation of movements involving those muscles; this effect occurs for both goal-directed and non-goal directed movements (Fadiga et al., 1995; Maeda et al., 2002).

Furthermore, the timecourse of MEPs follows the timecourse of the observed action, showing that the human motor system matches the individual, component movements of an observed action (Gangitano et al., 2001). Additionally, electrical stimulation to a nerve produces activation (twitching) in monosynaptically-connected muscle fibers, called the H-reflex. Baldissera et al. (2001) elicited H-reflexes from flexor finger muscles while subjects viewed a hand either opening or closing. Activation of the flexor muscles was greater when subjects observed a hand *opening*, which is the opposite of what occurs during actual hand-opening execution (flexors close the hand) and also opposite to the resonant excitability that occurs through stimulation at the level of the cortex (i.e., the TMS experiments above). This implies that motor resonance in the brain is somehow inhibited in the periphery. Because the H-reflex is known to be monosynaptic, this indicates that this inhibition occurs at the level of the spinal cord.

Human electrophysiology experiments have also found a shared basis for action execution and observation. Humans show suppression of sensorimotor cortical EEG rhythms during both action observation and execution, measurable with either EEG or MEG (Pineda, 2005; Hari, 2006). This occurs during observation of facial expressions as well as both transitive and intransitive limb movements and is distributed somatotopically over sensorimotor cortex according to the body part being observed (Muthukumaraswamy and Johnson, 2004; Muthukumaraswamy et al., 2004, 2006; Oberman et al., 2005; Moore et al., 2011). The effect is stronger for reach-to-grasp actions that are directed toward an object than those that are not (Muthukumaraswamy and Johnson, 2004; Muthukumaraswamy et al., 2004).

These types of TMS, EEG, and MEG experiments have not been performed in macaques, but single-cell recordings show that mirror neurons in ventral premotor area F5 and inferior parietal areas PF/PFG respond to both the execution and observation of similar movements, including both manual actions and orofacial movements (Gallese et al., 1996; Rizzolatti et al., 1996; Ferrari et al., 2003). However, macaque mirror neurons only respond to observed manual actions which are object- or goal-directed; they do not respond to observed mimed (intransitive) actions (Gallese et al., 1996; Rizzolatti et al., 1996; Ferrari et al., 2003). The human homologues of macaque F5 and PF/PFG are Brodman areas 44 and 40 (Rizzolatti and Craighero, 2004). In neuroimaging experiments, these regions are active during observation and execution of similar actions in a somatotopic manner (Buccino et al., 2001). Motor contagion in humans has been proposed to rely on a mirror system homologous to that in macaques (Blakemore and Frith, 2005). If this is true, then motor contagion and motor interference should occur in macaques (as well as any other species that have a mirror system), although to our knowledge this has not been tested.

In addition to the reflexive phenomena described above, individuals can also copy each other's behavior in a less automatic, more controlled manner. Many species are capable of using observational learning to copy another's goal-directed action. Rats can learn to run a maze by observing another rat (Zentall and Levine, 1972). Some birds socially learn each other's songs (Zentall, 2004). Guppy fish can socially learn foraging innovations (Laland and Reader, 1999). Wild macaques learn to wash sand off sweet potatoes by watching other macaques (Kawamura, 1959). Both capuchin monkeys and chimpanzees learn to use tools by watching conspecifics (Fragaszy and Visalberghi, 1989; Inoue-Nakamura and Matsuzawa, 1997).

Undoubtedly, not all of these phenomena need to be understood as involving reflective processing. When considering the impressive variety of social learning across species, it is important to recognize that the same general function—copying another's behavior—can result from different psychological and neurophysiological mechanisms in different species. Various schemas exist for categorizing different types of social learning behavior [e.g., (Whiten et al., 2004; Zentall, 2006)]. In general, the types of social learning behavior that are most widespread across species do not involve a representational understanding of the goal behind an observed action; for example, observers' attention may be drawn to particular objects or locations in the environment, facilitating their own independent discovery of how to produce an action involving that object (stimulus enhancement); they may learn about the positive or negative value of an object or event (valence learning); or they may reflexively copy aspects an observed action's movements without reflective understanding of its goal (mimicry). Many of these behavioral phenomena may occur reflexively, without representational understanding of the observed action's goal.

Forms of controlled social learning that involve an understanding of the observed goal are more rare, but are well-studied in primates. Most primate social learning is classed as emulation (copying an action's goal or result but not specific movements or methods) rather than imitation (copying both the goal and methods) (Whiten et al., 2009). While some studies report imitation in other species [e.g., chimpanzees: (Hayes and Hayes, 1952; Custance et al., 1995; Horner and Whiten, 2005); marmosets: (Voelkl and Huber, 2000)], none of these species use it so profusely and complexly as humans. In particular, a decades-long body of behavioral research describes a bias toward emulation in chimpanzees, and a bias toward imitation in humans (Whiten et al., 2009). For example, in one task (Horner and Whiten, 2005), the experimenter demonstrates a complex series of actions that open a puzzle box (pulling levers, pressing buttons, etc.). When the puzzle box is opaque and the relationship between these maneuverings and the opening of the box is not perceptible, both chimpanzees and human children copy these actions with high fidelity. However, if a transparent box is used, it becomes obvious that some of the demonstrator's actions do not contribute to opening the box. Chimpanzees dispense with these useless actions and use the most efficient method to open the box. Human children, on the other hand, persist with these actions, even when instructed not to reproduce any “useless” or “silly” actions, and even when they verbally report that they understand that they are useless (Lyons et al., 2007). This is termed “overimitation,” and it is even stronger in adults than in children (McGuigan et al., 2011).

Developmentally, copying of goal directed actions emerges in humans improves over the first two years of life, and in chimpanzees during the first four years (Inoue-Nakamura and Matsuzawa, 1997; Elsner, 2007; Elsner et al., 2007). Human infants are more likely to reproduce actions that have goals than those that do not (Elsner, 2007), and when preschool children copy a goal-directed movement, they tend to use movements that are less congruent with the demonstrator's than if there is no goal (Bekkering et al., 2000). It is interesting to note that motor interference effects are not observable until the age of four to five years (Marshall et al., 2010; Saby et al., 2011), suggesting that motor resonance, which would otherwise cause interference, may be somehow damped during the time that goal-directed copying is developing. However, children show electrophysiological correlates of motor resonance (mu suppression) as early as 6 months and seem to do so throughout development (Lepage and Theoret, 2006; Nystrom, 2008; van Elk et al., 2008). An important area of future research will be the developmental relationship of reflexive motor resonance phenomena with more controlled social learning phenomena.

To date, the neural correlates of goal-directed behavioral copying have only been studied in humans. In humans, two recent meta-analyses of functional neuroimaging studies on imitation found that it involves the homologues of the macaque mirror regions (Brodman areas 44 and 40), as well as broader regions of superior parietal lobe, inferior parietal lobe, dorsolateral prefrontal cortex, and premotor cortex (Caspers et al., 2010; Molenberghs et al., 2011). Lesions to either frontal or parietal regions can cause apraxia, a neuropsychological disorder of imitation (Goldenberg, 2009). While the macaque mirror system is activated by the observation of goal-directed actions, notably, monkeys do not imitate according to the definition above (copying both goal and method; Fragaszy and Visalberghi, 2004). However, macaques do recognize when their goal-directed actions are being imitated by a human experimenter (Paukner et al., 2005). Even accepting a looser definition of imitation, it is obvious that macaques' social learning is less profuse and less complex than humans'. Furthermore, the macaque mirror system does not respond to meaningless actions not directed at an object, e.g., mimed grasping, while the human mirror system does (Rizzolatti and Craighero, 2004). This suggests that species differences in the mirror system could be related to species differences in social learning.

One recent study examined the white matter connectivity of the mirror system in macaques, chimps, and humans (Hecht et al., 2012). In macaques and chimps, the bulk of the white matter within the mirror system connects temporal perceptual areas directly to the frontal mirror region and surrounding frontal areas. Since the frontal mirror region is thought to contain a “vocabulary of motor acts” where actions are coded according to their goals or results (Rizzolatti et al., 1988; Bonini et al., 2009), this pathway might underlie macaques' and chimps' bias toward copying an action's results over its movements. In humans, relatively more white matter in the mirror system passes through parietal cortex. Since the parietal mirror region is thought to perform sensorimotor mapping of the spatial and temporal details of observed and executed movements (Rozzi et al., 2008; Bonini et al., 2009), this increased connectivity might allow humans to map observed actions onto their own motor systems with greater kinematic detail, and could be related to our propensity for “overimitation.”

Taken together, research on phylogeny, development, and neural activation suggests that self-other mapping in the somatomotor domain can occur via both reflexive and reflective processes. A reflexive mechanism is in place very early whereby observed movements are automatically reproduced. After a short period—days, weeks, or months depending on the species (with unknown implications of this difference)—an inhibitory process comes online and this automatic mimicry disappears. In human adults, this inhibition seems to be mediated by the spinal cord, perhaps leaving the brain free to mirror observed action uninhibitedly (Rizzolatti and Craighero, 2004). This direct, low level self-other matching mechanism is thought to result from simple Hebbian synaptic potentiation during development: an individual's own action causes motor and visual neurons to “fire together,” increasing the chances that they will eventually “wire together,” so that after repeated co-activation, activation in one neuron alone can cause activation in the other, creating neurons that activate in response to observed, unexecuted action (Keysers and Perrett, 2004; Brass and Heyes, 2005). Such a mechanism should be widespread across phylogeny, might account for the development of premotor/parietal mirror neurons as well as other, heterogeneous cell types, and might account for motor contagion and mimicry across various species.

On the other hand, a reflective mechanism allowing the reproduction of goal-directed actions emerges later in development and is more limited across phylogeny. In humans, it involves some of the same neural substrates as reflexive motor resonance, as well as other regions more commonly associated with reflective processing, like dorsolateral prefrontal cortex and superior parietal cortex (Caspers et al., 2010; Molenberghs et al., 2011; Koenigs et al., 2009; Barbey et al., 2012a,b). A sub-distinction can be made between copying actions' results versus movements; humans focus on copying movements, while chimpanzees and other primates focus on copying goals. This difference in behavior may be the result of an underlying difference in neural responsivity (whether the mirror system can respond to intransitive action), which itself may be a result of a difference in white matter connectivity (the amount of connectivity with parietal cortex) (Hecht et al., 2012). The idea that copying results and copying movements are semi-dissociable processes is supported by clinical evidence. Goldenberg (2009) argues that lesions to frontal cortex in humans impair imitation of goal-directed actions, while lesions to parietal cortex impair imitation of non-goal-directed, meaningless actions. Furthermore, non-goal-directed imitation may be specifically impaired in autism (Gowen et al., 2008). (Paulus et al., 2011) suggest that developmentally, motor resonance is necessary but not sufficient for social learning of goal directed actions. This holds across phylogeny: reflexive motor resonance and mimicry are seen across a wide variety of species, and seem to be necessary but not sufficient for the development of social learning involving a reflective understanding of observed goals, which is more rare across phylogeny.

### Self-other matching in the perceptual domain: eye movements and cognition about perception

Individuals can match their own visual perception or attention to that of another by following gaze direction (Emery et al., 1997). It is easy to see how gaze following is a broadly adaptive trait—if something has drawn my conspecific's attention, it likely deserves my attention as well, since we share food sources, predators, prey, and potential mating partners. Bringing one's own perception into congruence with another individual's can also serve as first step toward bringing behavior into congruence. Therefore, it is not surprising that this basic behavior occurs automatically across the animal kingdom, in various species of reptiles, birds, and mammals.

In its simplest form, gaze following is tested by having the subject view a conspecific or human experimenter looking up, down, or to the side, and measuring whether the subject performs a congruent adjustment in visual attention. This test is passed by tortoises, a variety of birds, domestic goats, dogs and wolves, and a variety of primates (Bugnyar et al., 2004; Schloegl et al., 2008; Loretto et al., 2009; Rosati and Hare, 2009; Wilkinson et al., 2010; Kehmeier et al., 2011; Range and Viranyi, 2011; Teglas et al., 2012). Some species, such as macaques (Emery et al., 1997), only follow shifts in head or whole body orientation, while others, such as chimpanzees (Tomasello et al., 2007), can follow shifts in eye gaze alone. Humans' white sclera make our eye movements more apparent than other species', who have darker sclera; this is thought to be a contributing factor in our ability to follow eye movements (Tomasello et al., 2007; Rosati and Hare, 2009).

In a more complex version of this task, the demonstrator individual looks toward an object that is occluded from the subject's view by a barrier. Animals that can pass this task are said to follow gaze “geometrically” and are inferred to have some referential understanding of the content of the demonstrator's perception—i.e., that the demonstrator is “looking *at*” a particular thing. Animals that fail this task are taken to lack the ability to take the visual perspective of others (Rosati and Hare, 2009). Species currently known to follow gaze geometrically include a subset of those above: spider monkeys and capuchins (Amici et al., 2009), chimpanzees, bonobos, and gorillas (Okamoto-Barth et al., 2007), dogs (Teglas et al., 2012), wolves (Range and Viranyi, 2011), rooks (Schloegl et al., 2008), and ravens (Bugnyar et al., 2004).

In a yet more complex task, perspective-taking is studied in humans and great apes using tasks that test the subject's ability to know that another individual does not know something that the subject does. For example, in the Sally-Anne test (Baron-Cohen et al., 1985), Sally places a toy in her basket and then leaves the room. Anne then enters the room and moves the toy. The subject is asked where Sally will look for her toy when she returns. This measures whether the subject has “theory of mind,” or the ability to attribute mental states or perspectives to others which are separate from one's own (Premack and Woodruff, 1978). Thus it is an explicit measure of a reflective process. However, there is evidence that implicit processing is also involved in this task. Both human adults and children are less accurate at judgments about their own visual perspective when there is another person present with a different physical perspective, suggesting that we reflexively map what others can see and that this uses the same cognitive machinery as awareness of what we can see (Samson et al., 2010; Surtees and Apperly, 2012). Human infants look longer at the correct answer in a Sally-Anne test before they can produce a correct explicit verbal response, suggesting that they have implicit awareness of others' perceptual knowledge (Clements and Perner, 1994). Various experiments suggest that chimpanzees are able to take the perspective of others (Povinelli et al., 1990; Hare et al., 2001; Brauer et al., 2007; Krachun and Call, 2009; Krachun et al., 2009). For example, in one study (Hare et al., 2001), subordinate chimpanzees preferred to approach food behind a barrier, so that a dominant chimpanzee could not see.

The complexity of gaze following behavior changes across development, and this differs between species. In humans, gaze following emerges between 3–18 months (Scaife and Bruner, 1975; Carpenter et al., 1998; Corkum and Moore, 1998). In rhesus macaques, it begins to emerge around 5.5 months; in chimpanzees, between 3–4 years (Rosati and Hare, 2009). At first, infants follow head movements but not eye movements alone, and continue to follow a demonstrator's repeated gazes toward an information-less target (such as a blank ceiling). This suggests a lack of understanding that eyes are the mechanism of perception, and that gaze following behavior is relatively inflexible, automatic, and not affected by learning. Later, infants begin to follow eye movements alone, and later still they can inhibit repeated gaze-follows to a meaningless target. The ability to follow gaze geometrically emerges around this time. This pattern of development is similar in wolves, macaques, chimpanzees, and humans (Scaife and Bruner, 1975; Carpenter et al., 1998; Corkum and Moore, 1998; Ferrari et al., 2000; Rosati and Hare, 2009; Range and Viranyi, 2011).

The neural basis of gaze following has been studied in humans and macaques. In humans, neuroimaging experiments have implicated the superior temporal sulcus, cuneus, inferior parietal lobule, and intraparietal sulcus in perceiving others' looking direction (Puce et al., 1998; Wicker et al., 1998; Hoffman and Haxby, 2000; Pelphrey et al., 2003, 2004; Materna et al., 2008). Superior temporal sulcus is involved in encoding intentions related to gaze (Pelphrey et al., 2003), while intraparietal sulcus may be related to shifts in one's own visual attention regardless of social context (Materna et al., 2008). In macaques, cells in superior temporal sulcus respond to different angles of head orientation (Perrett et al., 1991). Cells in area LIP of the intraparietal sulcus fire both when the monkey looks in the cell's preferred direction and when another monkey looks in the same direction (Shepherd et al., 2009). A second population of cells in this area was suppressed by the observation of other monkeys' gaze. Interestingly, most of F5 mirror neurons are tuned to a particular visual perspective for observed grasping movements, suggesting a role for perspective in the somatomotor self-other matching system (Caggiano et al., 2011).

Considering the neural and behavioral research together across phylogeny, some patterns emerge. There are no species that are capable of following eye movements alone but not head movements, or head movements but not whole body movements. Developmentally, across species, the ability to follow eye movements alone emerges after the ability to follow head or body movements. Additionally, there are no species that follow gaze behind a barrier but not into empty space, and following gaze into empty space always emerges in development before following gaze around a barrier. The ability to follow gaze geometrically co-emerges with the ability to *not* follow repeated gazes toward an informationless target, such as a blank ceiling. Thus it appears that there are two fairly discrete components to gaze following: an early-developing, egocentric, automatic one, and a later-developing, allocentric, controlled one that takes into account the referential information in the gaze.

It seems likely that these components might rely on at least partially separable neural substrates. Shepherd et al. (2009) suggest that LIP cells are involved in the reflexive mode of gaze following. Similarly, Pelphrey et al. (2003) suggest that human intraparietal sulcus is concerned with egocentric mapping of spatial attention. This suggests the hypothesis that the automatic, implicit mode of gaze following can be mapped to parietal cortex. We wonder whether Shepherd et al. (2009) second population of cells that were suppressed by observed gaze changes might serve to override this automatic “mirroring” of attention, and whether the onset of their inhibition during development might coincide with the onset of the ability to habituate to meaningless gazes. Conversely, Pelphrey et al. (2003) suggest that in humans, the superior temporal sulcus may be more involved with judging the intentionality of others' actions, and has been implicated more broadly in reflective social cognitive processes like theory of mind. Thus we can hypothesize that this region might underlie the referential understanding of the content of others' gaze.

### Self-other matching in the autonomic/emotional domain

In addition to the somatomotor and oculomotor domains, self-other matching also occurs in the autonomic domain. This can extend to very low-level functions, such as pupil size (Harrison et al., 2006, 2007, 2009) and respiration (Jeannerod and Frak, 1999; Paccalin and Jeannerod, 2000; Mulder et al., 2005; Kuroda et al., 2011). “Contagion” of autonomic states has been well studied across species in the domain of pain, fear, and anxiety. For example, geese have heart rate increases after viewing their mate in conflict (Wascher et al., 2010). Mice have stronger responses to pain after viewing another mouse in pain (Langford et al., 2006; Jeon et al., 2010; Jeon and Shin, 2011). Monkeys exhibit behavioral signs of fear when watching another monkey in fear, even when the observer cannot see the item that is feared (Mineka and Cook, 1993). Crying is contagious in human infants (Geangu et al., 2010). In adult humans, photographs of others in danger or pain induces a freezing postural response (Azevedo et al., 2005; Facchinetti et al., 2006).

Beyond simply “catching” the emotion of fear non-referentially, various species can learn *what* to fear by watching others through observational learning. For example, in an experiment with crows, adult crows were captured, banded, and released by human experimenters who wore distinctive masks. The offspring of these adult crows, who observed the masked experimenters' actions, later produced alarm calls to humans wearing the same masks, even though they had no interaction with the humans personally (Cornell et al., 2011). Similarly, monkeys can acquire fear of snakes after watching other monkeys' fearful interactions with snakes, without any personal experience with snakes (Cook and Mineka, 1989, 1990). When human adults observe others undergoing a panic attack after a conditioned stimulus, they show greater electrodermal responses and report more fear and anxiety for that stimulus (Kelly and Forsyth, 2007). In humans, observational learning of fear, like Pavlovian conditioning, subsequently produces increased skin conductance measurements in response to a masked (nonconsciously viewed) image, while simple verbal instruction that an item is dangerous does not (Olsson and Phelps, 2004). This suggests that observational learning of fear acts via a reflexive, implicit mechanism rather than a controlled, explicit mechanism.

Individuals of various species can also learn what *not* to fear by watching others. Attenuation of fear by observational learning has been reported in mice (Guzman et al., 2009), and extinction of avoidance behavior is facilitated by observational learning in rats (Uno et al., 1973). Monkeys that observe other monkeys behaving non-fearfully with snakes are less likely to acquire fear of snakes themselves, and overshadowing can also be achieved through observational learning in monkeys (Mineka and Cook, 1986; Cook and Mineka, 1987). Human children who see their mothers responding positively to a fear-inducing stimulus are less fearful of the stimulus (Gerull and Rapee, 2002; Egliston and Rapee, 2007). For human children learning to overcome a fear of swimming, swimming lessons are more effective when paired with observation of a non-fearful child swimming (Weiss et al., 1998).

Self-other matching for autonomic states seems to rely on the same neural structures that produce those states in the observer. In mice, observational fear learning is blocked by inactivation of the anterior cingulate or the thalamic pain nuclei (both regions involved in the experience of pain), but not thalamic sensory nuclei (Jeon et al., 2010). In humans, felt and seen pain activate anterior cingulate and anterior insula (Lamm et al., 2010). Felt and seen disgust also activate the insula (Wicker et al., 2003; Wright et al., 2004; Jabbi et al., 2008). The amygdala seems to be necessary for not only the experience of fear, but also the perception of fear in others—Adolph's famous patient SM, who suffered bilateral calcification of the amygdala, is both unable to experience fear personally and has difficulty attributing it to others (Adolphs et al., 1994; Feinstein et al., 2010).

Another example of automatic, reflexive self-other matching in this domain is facial expressions. As mentioned previously, orofacial movements are automatically imitated for a brief postnatal period in macaques, chimpanzees, and humans (Meltzoff and Moore, 1977, 1983; Heimann et al., 1989; Myowa-Yamakoshi et al., 2004; Ferrari et al., 2006, 2009a,b; Paukner et al., 2011), and adult orangutans rapidly mimic facial expressions during play (Davila Ross et al., 2008), but no other studies have assessed involuntary facial mimicry in adult animals. In adult humans, viewing another individual's facial expression causes rapid facial reactions, or brief, reflexive, low-intensity mimicry of the expression in one's own face, measureable with EMG (Dimberg and Thunberg, 1998). This occurs even when stimuli are presented to the blind hemisphere of patients with unilateral visual cortex lesions, so it does not require cortical awareness (Tamietto et al., 2009). Interfering with this ability reduces emotion detection accuracy—subjects are less accurate at naming happy facial expressions when holding a pencil in their mouth (Oberman et al., 2007), lesions to somatosensory cortex impair facial expression recognition (Adolphs et al., 2000), and Botox injections decrease emotion recognition across multiple expressions (Neal and Chartrand, 2011). Furthermore, the application of a restricting gel to facial skin, which increases feedback signals, increases emotion perception accuracy (Neal and Chartrand, 2011). This suggests that some part of this implicit, automatic mimicry is informational—i.e., facial feedback from the mimicked expression activates neural representations about the meaning of the expression. However, facial expressions, face-voice combinations, and body expressions all evoke similar EMG responses in the face, suggesting that humans also resonate with the affective meaning of expressions and not just the motor pattern (Magnee et al., 2007).

Motor resonance and contagion for facial expressions seems to rely on some of the same mechanisms as motor resonance and contagion for somatomotor movements. While viewing facial expressions, neonatal macaques show mu suppression, thought to be an EEG index of mirror neuron activity (Ferrari et al., 2012). Adult macaques activate frontal mirror neurons during the observation of facial expressions (Ferrari et al., 2003). Human children (Dapretto et al., 2006) and adults (Molenberghs et al., 2011) activate inferior frontal gyrus, the homologue of macaque F5, during the observation of facial expressions, and also show mu suppression during facial expression observation (Oberman et al., 2005; Moore et al., 2011). Interestingly, infant macaques who imitate facial gestures have more developed reaching-grasping behavior and fine motor control in the hand than their conspecifics who do not, providing further evidence that this phenomenon is linked to motor resonance in the somatomotor domain (Ferrari et al., 2009b).

Yawns are a specific example of a contagious facial expression that is contagious in several species. In addition to humans, macaques (Paukner and Anderson, 2006), gelada baboons (Palagi et al., 2009), chimpanzees (Anderson et al., 2004; Campbell et al., 2009; Campbell and de Waal, 2011), and dogs (Joly-Mascheroni et al., 2008; Harr et al., 2009) also experience contagious yawning. In humans, viewing others' yawns activates precuneus, posterior cingulate, and superior temporal sulcus, all regions that have been associated with “higher-level” forms of social cognition (Platek et al., 2005; Schurmann et al., 2005). Platek (2010) notes that individual humans who are more susceptible to contagious yawning tend to be better at higher-order social cognitive measures like theory of mind processing and self-face recognition, and suggests that yawn contagion may be an evolutionarily old processes that became the basis for these more complex forms of social cognition.

In addition to self-other matching of autonomic states and facial expressions, others' emotions can also be matched in a more explicit, reflective manner. Preston and de Waal (2002) use the term “cognitive empathy” to describe a referential understanding of another's emotional state. Several studies show a link between reflective and reflexive self-other matching of emotion. Subjects who score high in emotional empathy scales have stronger facial mimicry for observed emotions, while low-empathy subjects activate facial muscles incongruent with the observed expression—e.g., “smiling” when seeing an angry face (Sonnby-Borgstrom, 2002). Similarly, high-empathy subjects show greater contagion for pupil size (Harrison et al., 2007). Autism and schizophrenia, both disorders which impair higher-order measures of empathizing, involve abnormal facial mimicry of observed facial expressions (McIntosh et al., 2006; Oberman et al., 2009; Varcin et al., 2010) and a reduction in yawn contagion (Haker and Rossler, 2009; Helt et al., 2010). A better understanding of the interaction between reflexive and reflective forms of emotional self-other matching may provide new directions for treatment in disorders of social cognition, since some problems in higher level social cognition and emotional response might derive from deficits in lower-level, reflexive self-other matching systems.

Another broad area of inquiry for future research is the interaction between self-other matching in the emotional domain with self-other matching in other domains. These interactions undoubtedly exist. For example, in the motor domain, as noted earlier, mimicry of postures, mannerisms, facial expressions, and behaviors increases liking, smoothes social interactions, and is more common in empathic people the chameleon effect (Chartrand and Bargh, 1999; Paulus et al., 2011). This brings up the interesting question of whether targeting or training self-other matching in the somatomotor domain (or another domain) might improve self-other matching in the emotional domain. Given that something like the chameleon effect seems to occur in capuchin monkeys, since monkeys prefer to interact with humans who imitate them (Paukner et al., 2009), research on this topic in other species might be useful for understanding it in our own.

## General discussion and conclusion

In this review, we have aimed to provide specific examples of how reflective processes are related to reflexive processes in self-other matching across species in three specific domains—in the motor domain (somatomotor movements), in the perceptual domain (eye movements and cognition about visual perception), and in the autonomic/emotional domain. Many unanswered questions remain; we have highlighted a few specific questions, with some potential ways to address them, in Table 2. Despite these unanswered questions, taking a broader perspective and considering these domains together, several patterns emerge.

**Table 2:** **Some unanswered questions for future research, with some suggestions for ways to address them**.

**General questions**	To what degree does self-other matching across domains rely on a common or shared mechanism?
	Is Hebbian learning during early development a general mechanism for self-other matching across domains? If so, can we find some sort of reflexive self-other matching in any organism that has Hebbian learning and a basic ability to perceive the behavior of conspecifics?
	Are there any experience-independent (hardwired) mechanisms for self-other matching?
**Motor domain**	The period of automatic mimicry of facial expressions last longer in humans than chimps, and longer in chimps than macaques. Is this relevant to adult species differences in social cognition? To address this question, we will first need to understand how neonatal mimicry impacts behavioral and neural development within these species
	Does automatic mimicry of facial expressions occur in non-primate mammals, reptiles, and birds? This might be studied with high-resolution video analysis of naturalistic social interactions
	Does the “chameleon effect” play a role in naturalistic social interactions in non-human species? If so, what is the neural mechanism? Following Paukner et al. (2011), this might be tested by experimentally manipulating whether an animal's behavior is copied and measuring ensuing social responses. Related neural activations might be mapped with FDG-PET (Rilling et al., 2007; Parr et al., 2009)
	Does motor resonance occur at low level, below the threshold for overt mimicry, in non-human animals? This might be studied with motor interference tasks, mu suppression of the EEG during observed movement, or the spinal H-reflex
	Mirror neurons have been found in macaques, rodents, and birds. This suggests that they likely exist in phylogenetically intermediate species. What other animals have mirror neurons, where are they, and how do they function?
	In humans, is motor resonance selectively damped during the time that children are learning to copy the goals of actions? This could be addressed with longitudinal studies mapping the time course of neonatal mimicry, motor contagion, goal-directed imitation, and motor interference within individual children
	Do humans have unique neuroanatomy or neural responses underlying our unique capacity for imitation and overimitation? Following Hecht et al. (2012), this can be accomplished with comparative neuroscience research
**Perceptual domain**	What is the role of perspective-taking in self-other matching in the somatomotor domain?
	How is the developmental stage of automatic gaze-following overridden? Does it coincide with the physiological development of inhibitory mirror neurons for gaze direction (Shepherd et al., 2009)?
	Are separate neural systems involved in automatic, reflexive gaze following and reflective, referential understanding of the content of others' visual perception?
**Autonomic/emotional domain**	What emotions are “contagious” in other species? Does this differ across species? This could be tested through naturalistic observation or laboratory-contrived situations that ensure that the observer's reactions cannot be attributed solely to own emotional response to the stimulus
	Do adult non-human animals show rapid facial reactions for observed facial expressions, or for bodily expressions of emotion? This could be measured with facial (or body) EMG
	If so, does self-other matching for facial/bodily expressions of emotion contribute to emotion understanding in these other species? This could be measured by training animals to do an explicit task on emotion identification (e.g., match to sample), interfering with mimicry similar to Oberman et al. (2007), and measuring changes in accuracy
	Following Platek (2010), why are human individuals who are more susceptible to contagious yawning better at measures of higher-order social cognition? More broadly, what is the relationship between low-level emotion/autonomic contagion and these more reflective functions?
	Can we treat dysfunctions in these more reflective functions by targeting underlying, reflexive functions?
	How does self-other matching in the emotional domain interact with self-other matching in other domains? Can we treat dysfunctions in emotional self-other matching by targeting self-other matching in other domains?

First, in each of these domains, there are early-developing, automatic processes that rapidly match the observer's state to others'. These could emerge based on a simple Hebbian mechanism, as individuals learn associations between observable effects and internal states within the context of their own behavior. As these associations are solidified, observation of only the process's effect (a fearful expression or the perception of an arm movement) can activate representations of the internal state that causes it (the emotion of fear or the motor representation of the arm movement). Since Hebbian learning is a common feature of nervous systems, seen even in mollusks, this type of self-other matching is likely widespread across the animal kingdom.

Second, more complex forms of self-other matching in each domain emerge later in development and are less prevalent across phylogeny. They involve some of the same neural substrates as their related lower-level processes, as well as other neural systems associated with representational thought. The function of the lower-level processes can impact higher-level processes. For example, paralysis of one's own facial muscles impairs recognitions of others' facial expressions (Neal and Chartrand, 2011). In general, these higher-level functions seem not to be present in species that lack the underlying lower-level functions—e.g., to date there are no species that are capable of geometric gaze following but not the simpler form of automatic gaze following into empty space. Many of these higher-level functions are uniquely developed in humans, and some may even be completely unique to humans. However, the longer that comparative psychology investigates which behaviors are uniquely human, the more once-unique functions are found in other species.

Third, the proper function and development of the lower-level systems is often critical for the proper function and later development of the higher-level systems. Because these higher-level functions like imitation, perspective taking, and empathy are more immediately observable and salient, social cognitive deficits are often attributed to dysfunctions in these higher-level functions, but it is important to also address whether there may be a less obvious deficit in an underlying lower-level function. For example, autism was once accepted as primarily a disorder of theory of mind (Baron-Cohen et al., 1985). More recent research, though, has shown that high-functioning autistic individuals can pass tests of theory of mind, albeit using different mechanisms. Current research is increasingly pointing toward cascade effects where early disruptions in lower-level social processes cause derailments of later-developing, higher-level processes. For example, early abnormalities in gaze following may underlie later deficits in perspective taking (Elsabbagh et al., 2012); abnormalities in motor resonance for body movements may lead to deficits in imitation (Gowen et al., 2008); and abnormalities in facial expression mimicry may be related to difficulties with empathy (McIntosh et al., 2006; Oberman et al., 2009).

A comparative, evolutionary approach highlights the role of these underlying, lower-level processes because it frames neural and psychological systems in a way that emphasizes continuity. As evolution produces organisms of increasing complexity, new functions must be integrated into the framework of pre-existing, simpler functions, like new heating systems being grafted onto old ones in an apartment building. An understanding of the normal or disordered function of the new systems would be incomplete without an understanding of the underlying, older systems, and of how the new are related to the old. Thus, our understanding of the psychological and neural mechanisms of self-other mapping, other forms of social cognition, and other functions in general in our own species can be informed by considering the layered complexity these functions in other species.

Of course, it is obvious that there are some things about human behavior and the human brain that are “special.” Some human behaviors or neural features may not have easily identifiable correlates in other species (although we argue that most probably do, to some extent). A comparative perspective can also inform understanding of behavioral abilities that only human have: they must rely on aspects of neural organization that are unique to humans. Unique neural features can only be mapped to unique behavioral features if we have a firm understanding of which neural and behavioral features are shared with other species.

### Conflict of interest statement

The authors declare that the research was conducted in the absence of any commercial or financial relationships that could be construed as a potential conflict of interest.
